# Management of Patients with Recurrent and Metachronous Oligometastatic Prostate Cancer in the Era of PSMA PET

**DOI:** 10.3390/cancers14246194

**Published:** 2022-12-15

**Authors:** Ali Sabbagh, Osama Mohamad, Katie E. Lichter, Thomas A. Hope

**Affiliations:** 1 Department of Radiation Oncology, University of California San Francisco, San Francisco, CA 94158, USA; 2 Department of Urology, University of California San Francisco, San Francisco, CA 94143, USA; 3 Department of Radiology and Biomedical Imaging, University of California San Francisco, San Francisco, CA 94143, USA

**Keywords:** prostate cancer, PET PSMA, radiotherapy, oligometastasis, biochemical recurrence

## Abstract

**Simple Summary:**

Prostate-specific membrane antigen positron emission tomography (PSMA PET) is a modern imaging modality used in the management of patients with prostate cancer with improved accuracy in detecting lymph nodes and distant disease spread. In this paper, we discuss how the increasing use of PSMA PET is changing clinical management in patients with prostate cancer, specifically those previously treated for localized disease and now presenting with recurrence or low-volume metastatic disease spread. We also discuss how PSMA PET is affecting clinical trial design and interpretation. More clinical trials are needed to investigate whether the use of PSMA PET translates into improved patient survival or quality of life.

**Abstract:**

Prostate-specific membrane antigen (PSMA) positron emission tomography (PET) scans have higher sensitivity and specificity for detecting lymph nodes or metastatic disease relative to conventional imaging in prostate cancer staging. Since its FDA approval and incorporation into treatment guidelines, the use of PSMA PET has increased in patients undergoing initial staging, those with recurrence after initial definitive treatment, and patients with metastatic disease. Although the early detection of metastatic lesions is changing disease management, it is unclear whether this impact on management translates into clinical benefit. This review will summarize evidence pertaining to the change in patient management due to PSMA PET use and will discuss the implications of PSMA PET on treatment decisions in prostate cancer, particularly in the settings of biochemical recurrence and metachronous oligometastatic disease.

## 1. Introduction

Prostate cancer (PCa) is the most common cancer and the second leading cause of cancer-related deaths in males in the United States with 248,530 new cases and 34,130 deaths in 2021 [1]. Treatment decisions for patients with PCa are dependent on several factors, including disease stage, grade, risk grouping, patient characteristics (e.g., life expectancy, performance status, comorbidities, etc.), patient preferences, and available resources. Initial cancer staging, staging in the setting of recurrence, and monitoring of response to systemic therapy in patients with metastatic disease rely on information provided by imaging. Magnetic resonance imaging (MRI) and transrectal ultrasound (TRUS) are used for the detection of intra-prostatic disease, extra-capsular extension, and seminal vesicle involvement. Computed tomography (CT) and bone scans have been the main imaging modalities used for the detection of metastases. Positron emission tomography (PET) imaging tracers, such as ^11^C-Choline and ^18^F-Fluciclovine, outperform FDG PET in the detection of nodal and osseous disease [2]. More recently, however, prostate-specific membrane antigen (PSMA)-based tracers have been found to be more accurate for the detection of prostate cancer [3,4,5,6,7,8]. PSMA is a type II transmembrane glycoprotein that is overexpressed in prostate cancer. PSMA PET relies on small molecules (^68^Ga-PSMA-11 or ^18^F-DCFPyL) that bind with high affinity to the extracellular component of PSMA [9]. ^68^Ga-PSMA-11 PET obtained FDA approval both for the detection of suspected metastatic lesions at initial staging and in the setting of biochemical recurrence (BCR) following curative therapy in December 2020. Following FDA approval, PSMA PET use was endorsed by the National Comprehensive Cancer Network (NCCN) and the Society of Nuclear Medicine and Molecular Imaging (SNMMI) guidelines [10,11].

**Figure 1:**
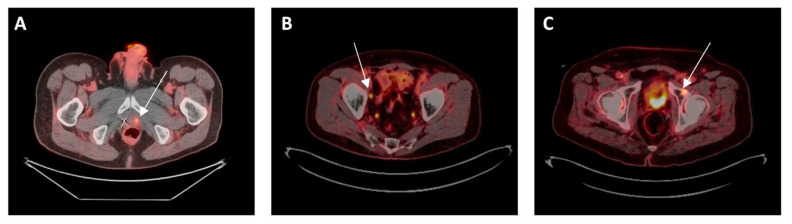
(**A**) Local failure after prior radiotherapy: 72-year-old man with intermediate-risk prostate adenocarcinoma treated with radiation to the prostate with biochemical failure 5 years later. PSMA PET CT showed uptake in left prostate but no regional or distant disease. He received salvage brachytherapy. (**B**) Regional failure after prior prostatectomy: 75-year-old man with high-risk prostate cancer treated with radical prostatectomy, with post-op PSA of 0.17, then treated with pelvic and prostate bed radiation. He again had rising PSA and PSMA PET CT showed uptake in one right pelvic lymph node but no local or distant disease. He received stereotactic radiotherapy to the lymph node. (**C**) Metachronous oligometastasis: 72-year-old man with intermediate-risk prostate adenocarcinoma treated with prostatectomy with undetectable PSA post-op, which then increased a few years later and PSMA PET CT showed uptake in left acetabulum. He received SBRT to the bone lesion without hormone therapy.

The influence of PSMA PET on the management of patients with PCa is undergoing extensive investigation. PSMA PET has higher sensitivity and specificity for detecting lymph node or metastatic disease and has a considerable impact on management decisions relative to conventional imaging [12]. Although PSMA PET is changing disease management, it is unclear whether this impact on management translates into clinical benefit; studies are ongoing to answer this question. In this review, we will summarize and discuss available evidence pertaining to the change in patient management due to PSMA PET use, specifically in patients previously treated for localized disease and now presenting with PSA recurrence, including those with metachronous oligometastasis (Figure 1). Given the differences in outcomes and possibly biology between patients with synchronous and metachronous low-volume metastatic disease [13], we will not discuss the use of PSMA PET in patients with newly diagnosed synchronous metastatic disease. We will also not discuss the impact of PSMA PET on newly diagnosed localized prostate cancer. Our goal was to write a narrative review of the literature, not a meta-analysis or a systematic review. We conducted our search on PubMed with the following strategy: prostatic OR “prostate cancer” AND “positron emission tomography’’ OR PET AND ‘‘prostate membrane specific antigen’’ OR PSMA AND management OR change OR impact. Abstracts were screened by the first co-authors and representative papers were included if relevant to the review’s subject. The search was limited to papers written in English and published between 2015–2022. Clinicaltrials.gov was used to select ongoing trials of PSMA PET. Tables were created to collect required information and to summarize the available evidence.

## 2. PSMA PET in the Setting of Biochemical Recurrence (BCR) without Evidence of Distant Metastasis

While several factors may affect PSA levels [14], an increasing PSA after definitive management usually indicates recurrence. In patients treated with radical prostatectomy (RP), BCR is generally defined as a detectable PSA level ≥ 0.2 ng/mL with a second confirmatory level [15,16]. EAU guidelines state that a PSA level > 0.4 ng/mL and rising after RP is a better threshold for the prediction of further metastases [17]. Patients with BCR after RP without distant metastasis are often treated with salvage RT (SRT) with or without hormone therapy. In patients treated with radiotherapy (RT), BCR is defined as a rise of ≥2 ng/mL above nadir PSA [18]. The management of BCR after RT begins with confirming the absence of disseminated disease (i.e., the recurrence is confined to the pelvis), with those patients meeting this criterion receiving local salvage treatment (to the prostate or to the pelvic lymph nodes). Patients with distant recurrence (after initial RP or RT) are started on androgen deprivation therapy (ADT) with or without hormone intensification. Traditionally, disseminated disease is ruled out using bone scan or CT. PSMA PET has better detection rates than conventional imaging [19]. A recent systematic review and meta-analysis, including 1309 patients with BCR from 16 studies, showed that the detection rate of ^68^Ga-PSMA PET increased with PSA. For the PSA categories 0–0.2, 0.2–1, 1–2, and >2 ng/mL, 42%, 58%, 76%, and 95% of the scans, respectively, were positive [12].

Three main studies have established the benefit of PSMA PET over conventional imaging in the recurrent setting. OSPREY is a phase 2/3 trial, which enrolled two cohorts of patients: cohort A included patients with high-risk prostate cancer, and cohort B included patients with metastatic or recurrent disease on conventional imaging. ^18^F-DCFPyL PET showed improvements in the specificity and positive predictive value when compared with conventional imaging [5]. CONDOR is a phase 3 study of patients with a median PSA level of 0.8 ng/mL after RP or RT and negative conventional imaging. ^18^F-DCFPyL PET improved disease localization in this cohort of patients [4]. Fendler et al. also prospectively assessed the accuracy of ^68^Ga-PSMA PET in patients with BCR after RT and/or RP and established high positive predictive value, detection rate, and inter-reader agreement for the localization of recurrent prostate cancer with PSMA PET [20]. It is, therefore, clear that PSMA PET has the potential of finding metastases or disease extensions that are not detected using conventional imaging. Table 1 summarizes a selected list of studies, which investigated the effect of PSMA PET on management decisions in patients with recurrent prostate cancer initially treated with RT or RP [21,22,23,24,25,26,27,28,29,30,31,32,33,34,35,36,37,38,39,40,41,42,43,44,45,46,47,48].

### 2.1. Biochemical Recurrence after Radical Prostatectomy

In patients with BCR after RP, detecting the presence and site of recurrence is crucial for RT planning. Intuitively, the presence of distant disease obviates the need for pelvic or prostate bed RT, and the presence of local failure or lymph nodes has implications for radiation field design, RT dose, and hormone therapy use and duration. Since most SRT is delivered at relatively low PSA levels (<1 ng/mL), conventional imaging is unlikely to add any valuable information for treatment planning, and thus most patients would receive SRT. The ability of PSMA PET to detect disease at low PSA levels prior to SRT offers a unique opportunity for personalized therapy. This has been shown in several studies. In their retrospective analysis, Bottke et al. evaluated the impact of PSMA PET on SRT planning in 76 patients with BCR after RP [21]. PSMA PET led to a change in therapeutic target volumes in 28% of patients. Similarly, Farolfi et al. looked at 119 patients with BCR following RP and found that RT planning was modified in 30% of patients [44]. These two studies and several others had a median PSA in the 0.2–0.3 ng/mL range, highlighting the fact that PSMA PET leads to significant change in post-RP radiotherapy management even at low PSA levels. This is extremely important given that SRT yields better oncologic outcomes when delivered at lower PSA levels (ideally <0.2–0.5 ng/mL) [49]. In summary, PSMA PET influences SRT planning anywhere between 28–76% of patients with biochemically recurrent prostate cancer following RP. Notably, the change in management is not only limited to SRT planning volumes and dose levels in the prostate bed or pelvis but also includes adding, stopping, and/or completely switching between treatment modalities. Patients who were previously thought to be eligible to receive SRT, for example, may be found to harbor distant metastasis and no longer receive local SRT. Such changes allow some patients to avoid unnecessary treatments while helping others receive more tailored therapy.

### 2.2. Biochemical Recurrence after Radiation Therapy

Compared to surgery, fewer studies have investigated the effect of PSMA PET on the management of patients treated initially with RT as the primary definitive treatment and presenting with BCR. In a prospective study, Liu et al. investigated the influence of PSMA PET compared to conventional imaging in managing patients with radio-recurrent prostate cancer. ^18^F-DCFPyL PET identified extra-prostatic disease in twice as many men and detected a site of recurrence in 87% of men, compared with 67% with conventional imaging. Furthermore, ^18^F-DCFPyL PET identified potentially actionable disease (prostate-only recurrence or oligometastatic disease) in 75% of men and changed the proposed management in 43% of men [32]. Afaq et al. evaluated the change in management following PSMA PET in 68 patients treated with RP and 32 patients treated with RT and found that 50% of the RT group had a change in their intended management after PSMA PET [50]. Barbaud et al. also studied patients with BCR after prior RP or RT who underwent PSMA PET imaging following negative Choline-11 PET and found that PSMA PET led to a change in the management in 73.8% of patients, with nearly 50% receiving radiation field adjustments [33]. Interestingly, several studies found that patients primarily treated with RT have a significantly higher rate of PSMA PET positivity, compared to patients treated with RP [34,36,50]. This higher rate of disease localization in patients who meet Phoenix criteria suggests that there may be a role for PSMA PET-based interventions at earlier PSA (before the nadir + 2 threshold) in patients with radio-recurrence. Fendler et al. and Hope et al. also reported that PSMA PET prevented additional tests from being performed and reduced the number of patients with unknown disease location by 50% [36,37]. Several other studies, including small prospective trials, also show that PSMA PET influences treatment management in patients with BCR [37,38,39].

To summarize, PSMA PET influences management in patients with BCR following both RT and RP despite the heterogeneity and retrospective nature of the studies and the variation in the definition of “treatment change” among the different studies. In a meta-analysis of 15 studies, including 1163 patients in the recurrent and definitive settings, the pooled proportion of management changes was 54% with half of these changes entailing a new treatment modality [51]. Although it is unknown whether this translates to better oncologic outcomes or improved quality of life, PSMA PET provides useful information to physicians, which affects their decision-making process. It is important to mention the recently published EMPIRE-1 study in which ^18^F-Fluciclovine-PET-based post-prostatectomy RT planning significantly improved survival free from biochemical recurrence or persistence, compared to conventional imaging [52]. Despite the limitations of the study, the results give hope that PSMA PET will favorably impact treatment outcomes, given its superior sensitivity and specificity over ^18^F-Fluciclovine-PET. Randomized trials looking at outcomes in patients whose management is planned with PSMA PET vs. standard of care imaging are ongoing to determine whether these changes are beneficial or not, namely in terms of survival and quality of life.

## 3. PSMA PET in the Setting of Metachronous Oligometastatic Disease

Oligometastatic prostate cancer (i.e., OMPC) includes patients who present with recurrence after initial definitive therapy with imaging showing limited metastatic involvement (i.e., metachronous), and patients presenting with de novo limited metastatic disease (i.e., synchronous). The distinction between synchronous and metachronous metastases is important as they may have different biologic and clinical characteristics. For the purpose of this paper, we will focus on metachronous oligometastatic disease. Whether OMPC is truly a distinct intermediate state between localized and widespread metastatic disease that is less aggressive and less likely to further metastasize is yet to be determined [53]. No consensus definition exists for OMPC. Traditionally, an accepted definition of OMPC is radiographic and is based on the number and location of metastatic lesions on conventional imaging [54]. The two most commonly referenced definitions of OMPC are derived from the “low volume” subgroup in CHAARTED [55], STAMPEDE [56], and GETUG-AFU 15 [57] or from the “non-high-risk” subgroup in LATITUDE [58]. However, the increasing use of PSMA PET and the increasing detection of small lesions that are otherwise not detected with conventional imaging are challenging these definitions. In addition to number and location, the genomic landscape of metastatic lesions also affect survival [59,60], and thus should also be considered in a more comprehensive definition for OMPC.

Two studies have defined the use of radiotherapy, specifically stereotactic body radiotherapy or SBRT, in patients with metachronous OMPC: STOMP and ORIOLE. The randomized phase II ORIOLE clinical trial compared outcomes of men with hormone-sensitive OMPC treated with metastasis-directed therapy (MDT) vs. observation [61]. MDT significantly decreased the progression of disease at 6 months relative to observation (19% vs. 61%, respectively). Although MDT was planned based on conventional imaging, a post hoc analysis revealed that patients who received consolidation to all detectable disease on PSMA PET had better progression-free survival (PFS) and distant metastasis-free survival than those who did not. These findings were validated in another prospective randomized phase II trial by Ost et al. (the STOMP trial), which looked at patients with recurrence after primary PCa treatment with three or fewer extracranial metastatic lesions on Choline PET (PSMA PET was not used). The median ADT-free survival was 21 months for the MDT group vs. 13 months for the surveillance group [62]. The benefit of MDT was maintained at longer follow-up with five-year data showing a 34% ADT-free survival for the MDT group vs. 8% for the surveillance group, as well as higher castrate-resistant prostate cancer (CRPC)-free survival (76% for MDT vs. 53% for surveillance). A combined analysis of STOMP and ORIOLE also showed that median PFS was prolonged with MDT, compared with observation [63].

Table 2 summarizes selected studies of PSMA PET staging and MDT in patients with OMPC [64,65,66,67,68,69,70,71,72]. The use of PSMA PET and MDT in the setting of OMPC has been investigated in a few prospective trials. In a single institution prospective study, Bowden et al. found that in a cohort of 176 patients, of whom 136 patients were initially staged with PSMA PET, median treatment escalation-free survival (TEFS) was 27.1 months. Patients staged with PSMA PET had a trend toward longer TEFS and lower treatment escalation rate at 2 years, although the difference was not statistically significant [64]. Similarly, Kneebone et al. found that biochemical disease-free survival was 11 months in patients with OMPC treated with MDT in 1–3 lesions detected on PSMA PET [65]. There were no local failures and toxicity was limited to grade 1 side effects. Several retrospective studies showcase the benefit of utilizing PSMA PET in patients with OMPC treated with MDT. Artigas et al. found an ADT-free survival of 74% at 2 years in 20 patients with hormone-sensitive OMPC. Biochemical response (defined as a PSA decrease by >50%) was seen in 70% of patients, and BCR-free survival was 53% at 2 years [66]. Guler et al. reported a PFS of 67% at 1 year for patients with castration-sensitive (CS) OMPC treated with MDT following PSMA PET. Notably, PFS was 0% for patients with castration-resistant (CR) OMPC, which they attributed to the more aggressive nature of CRPC [67]. Similar benefits were seen in other studies, including patients with castration resistance [68,69,70,71]. The case of castration resistance is particularly interesting given the upregulation of PSMA expression with the use of ADT, the possible association of PSMA expression with castration resistance, and the higher standardized uptake values (SUVmax) in patients with CRPC, compared to those with CSPC [73,74,75]. Finally, Deijen et al. retrospectively compared PSA response, PSA response duration, and ADT-free survival in patients with OMPC treated with PSMA PET- vs. Choline-11 PET-guided SBRT. PSMA PET led to improved ADT-free survival, as well as longer PSA response, compared to Choline-11 PET [72].

While the data are promising, these studies have several limitations. Namely, most of these studies are retrospective in nature, hence prone to a wide range of biases probably leading to an over-estimation of the effect size. Furthermore, patient and treatment characteristics are heterogeneous both among studies and within the same study. Caution is thus warranted in interpreting the results. Given all this information, PSMA PET potentially holds appreciable benefits in guiding MDT for patients with OMPC, and thus delaying the initiation of ADT or delaying the switching of systemic therapy at progression. Again, however, whether this has an impact on outcomes remains poorly understood and should be further investigated.

## 4. Discussion

Most of the current recommendations for the treatment of patients with prostate cancer are derived from older studies, before the widespread availability of PSMA PET. The increasing use of PSMA PET has caused a landscape shift and has led to new paradigms and disease presentations, which were not appreciated in the era of conventional imaging. Therefore, while findings are promising, it is still unclear how PSMA PET should change management and how to integrate these advances into modern clinical practice and future trial design. Interestingly, PSMA PET is having a significant impact on the interpretation of endpoints in clinical trials. For example, it is unclear how progression on PSMA PET should be exactly defined and whether it is clinically meaningful, especially when considering that PET-based metastatic progression happens before progression on conventional imaging. In patients enrolled on clinical trials, PSMA PET-based interventions may prolong conventional imaging-based metastasis endpoints and make interpretations of trial results difficult. Along the same lines, it is unclear whether progression on PSMA PET should lead to a change in therapy, such as adding or switching systemic therapies or adding MDT. Although a proposal for systemic therapy response–assessment criteria with PSMA PET has been published [76], more work needs to be done in this domain to connect the progression criteria with clinical meaningfulness.

While not discussed in this review, PSMA PET imaging is indicated per the NCCN guidelines in the initial staging of patients with unfavorable intermediate- and high-risk disease. PSMA PET could be used for dose escalation to avid lesions, as per the FLAME study (which used MRI rather than PSMA PET) [77,78], given the high correlation between PET avidity and tumor aggressiveness on histopathology [79,80]. Although results from the PRIMARY clinical trial showed improved sensitivity and negative predictive value for the detection of clinically significant cancer with PSMA PET compared to MRI [81], it is unclear what added advantage exists for using PSMA PET images alone or in combination with MRI images over MRI alone for focal boosting or even for focal prostate treatments. More studies are needed in this setting. In the recurrent setting, some patients with BCR after RP will have a change in their treatment plan, including the addition of a new treatment modality or a complete change in modalities, based on PSMA PET findings. This is true even at low PSA values (0.2–0.5 ng/mL). Whether this change in treatment planning will improve outcomes remains to be proven. Nonetheless, PSMA PET opens the door for more personalized therapy, as opposed to a “one-size-fits-all” approach. In patients with BCR after RP and with a negative PSMA PET done at an appropriate PSA level, salvage RT should be offered without delay and patients should be counseled about the low detection rate at low PSA and the overall low sensitivity of PSMA PET imaging [7]. A negative PET should not be used to postpone salvage RT. Patients with BCR after prior definitive treatment with PSMA positive nodes in the pelvis may benefit from pelvic radiotherapy (plus boost to avid nodes) and long-term ADT (with or without intensified hormonal therapy). In patients treated with prior RP, the additional treatment of the prostatic fossa with radiation is also indicated in most cases. Patients with BCR after definitive treatment and PSMA-positive retroperitoneal nodes require ADT (with or without intensified hormonal therapy). Radiotherapy could be delivered with full pelvic and para-aortic fields or with SBRT to the involved nodes. PSMA PET studies on the patterns of failure after initial definitive treatment will continue to define consensus guidelines for pelvic radiotherapy volumes [82,83]. Clinicians evaluating PSMA PET images, however, should be aware of the potential pitfalls of PSMA PET imaging, including an 8% chance of false positive readings (e.g., within a previously irradiated prostate) and an 8% chance of false negative readings (e.g., urine activity or small size lesions) [84]. Interestingly, the biopsy of PSMA-positive lesions is rarely done in clinical practice, but clinicians should assess findings cautiously, especially when PSA or SUV_max_ are low, when there is only one lesion on the scan (e.g., solitary rib lesion), or when there is an absence of CT correlates. Such findings should raise suspicion for a false positive.

The increased sensitivity of PSMA PET leads to stage migration as patients previously thought to be at a low stage are upstaged based on new PSMA PET findings. This leads to improved survival in both stages: survival in the lower stage improves as the more advanced patients are upstaged and survival in the higher stage also increases when favorable patients enter this group. It is unclear how to exactly deal with stage migration when deciding on treatments, especially in patients considered for clinical trials. For example, how do we manage patients with BCR after prior RP and negative conventional imaging but with one small distant lesion on PSMA PET? Is biopsy always indicated in these situations? Should these patients receive salvage RT to the pelvis, or should they be treated with systemic therapy? If so, what is the most appropriate systemic therapy? Additionally, should they receive MDT? Finally, could such patients be enrolled on a trial of salvage radiotherapy that does not mandate molecular imaging? Currently, the clinical trial NRG GU-008, which randomizes patients with nodal involvement after prostatectomy to salvage radiation and long-term ADT with or without Apalutamide, excludes patients with M1 disease on molecular imaging. Similarly, it is unclear how to manage definitive patients with PSMA PET findings which are not corroborated by conventional imaging. Interestingly, the currently enrolling NRG GU-009 and NRG GU-010 allow patients who have bone metastases established only by Fluciclovine, Choline, or PSMA PET but are not definitive on bone scan or NaF PET to enroll. Finally, it is not clear how to specifically treat patients with metachronous OMPC with bone lesions per PSMA PET. Options include ADT alone, ADT with additional androgen receptor (AR)-targeted agents, MDT alone, MDT and ADT, or MDT and ADT plus additional AR-targeted agents. Currently, there is extreme heterogeneity in how these patients are being treated clinically, including patients progressing on clinical trials, which will make the future interpretation of data difficult. NRG GU-011 is trying to answer one part of this question.

This review has several limitations. First, we did not follow a systematic approach for the selection of papers as in a systematic review or meta-analysis. This means that there is the potential for selection bias. Second, we did not formally evaluate bias or heterogeneity. Third, the cohorts of patients included are not homogeneous. Although each section deals with a general presentation of disease (e.g., biochemical recurrence, OMPC, etc.), each study had its own inclusion and exclusion criteria, as well as its own definition of recurrence or OMPC. Thus, caution is warranted in drawing conclusions. Fourth, most of the included studies are retrospective in nature, and thus are inherently prone to certain biases, such as overestimation of effect size. Although a more systematic review should be conducted in the future, our work nonetheless provides readers with a summary (both quantitative and qualitative) of a large body of evidence relating to the effect of PSMA PET on management decisions. Finally, while we group all PSMA PET imaging modalities together, it is important to discuss the advantages and limitations offered by PSMA PET CT versus PSMA PET MRI. PSMA PET MRI combines the molecular data from PET with the superior anatomic details of the MRI. Although PSMA PET MRI shows good diagnostic performance, there is no clear data showing its superiority over PET CT [85]. PET CT scans are more widely available, less expensive, and the scans are faster and more comfortable for the patient but PET MRIs have better soft-tissue contrast.

## 5. Future Directions

As previously discussed, most of the available literature is retrospective in nature, severely limiting reliable conclusions. Several prospective trials are currently underway evaluating the role of PSMA PET imaging in the management of prostate cancer at initial staging, recurrence, and in (oligo)-metastatic disease. Importantly, many of these studies are comparing oncologic outcomes between standard of care vs. molecular imaging. A selected list of ongoing trials is presented in Table 3. NCT04794777 and NCT03582774 are randomized, open label, clinical trials that compare outcomes of patients presenting with BCR after RP, randomized to either standard SRT vs. individualized therapy based on PSMA PET. NCT04983095 is an open label, multi-center, randomized study that will look at patients with OMPC treated with SBRT and standard of care therapy vs. standard of care only. The PILLAR study (NCT03503344) is investigating the efficacy of apalutamide with or without MDT in treating participants with oligometastatic CRPC, with undetectable PSA at 6 months as the primary endpoint. Furthermore, other PSMA tracers are also being investigated, both in terms of metastasis detection rate and their effect on patient management. For example, NCT04978675, a prospective trial conducted at MD Anderson Cancer Center, is assessing the use of rh PSMA 7.3 PET/MRI in detecting recurrent prostate cancer, as well as its effect on salvage RT planning. Finally, as PSMA-targeted radioligand therapies (e.g., ^177^Lu-PSMA-617) show clinical benefit, the underlying resistance mechanisms and the role of PSMA PET imaging as a biomarker for patient selection will need to be further investigated in future studies [86,87,88,89].

## 6. Conclusions

In conclusion, PSMA PET provides many answers and important information for clinicians when it comes to the management of patients with prostate cancer even at low PSA values where traditional imaging modalities are completely inadequate at detecting malignant lesions. However, PSMA also raises many questions, which remain unanswered. Larger randomized trials are needed to truly validate the effect of PSMA PET on patient outcomes in different disease settings.

## Figures and Tables

**Table 1:** Selected studies on the impact of PSMA PET on treatment planning in patients with recurrence after definitive therapy.

Lead Author	Year	N	RP	RT	Median PSA (ng/mL)	PSA Limit (ng/mL)	PSMA Positivity	Treatment Planning Change (% of All Patients in Cohort)	Major Changes	Minor Changes	Radiotracer and Equipment
Albisinni et al.	2017	131	106	25	2.2	None	75%	76%	NA	NA	^68^Ga-PSMA-PET/CT
Barbaud et al.	2018	42	32	28	2.56	None	80.90%	73.80%	NA	NA	^68^Ga-PSMA-PET/CT
Bashir et al.	2019	28	28	0	0.22	0.5	60.70%	42.80%	NA	NA	^68^Ga-PSMA-PET/CT
Bianchi et al.	2019	276	276	0	0.72	None	47.50%	66.60%	64.10%	2.50%	^68^Ga-PSMA-PET/CT
Bluemel et al.	2016	45	45	0	0.67	None	53.30%	42.20%	NA	NA	^68^Ga-PSMA-PET/CT
Boreta et al.	2019	125	125	0	0.4	2	53%	30% **	NA	NA	^68^Ga-PSMA-PET CT or MRI
Bottke et al.	2021	76	76	0	0.245	0.5	54%	28%	17%	11%	^68^Ga-PSMA-PET/CT
Calais et al.	2018	270	270	0	0.48	1	49%	48.50%	19%	29.50%	^68^Ga-PSMA-PET/CT
Cerci et al.	2021	1004	780	224	1.55 (mean)	None	65.13%	56.80%	NA	NA	^68^Ga-PSMA-PET/CT
Deandreis et al.	2020	121	121	0	0.66	1.5	36.30%	29.70%	NA	NA	^68^Ga-PSMA-PET/CT
De Bari et al.	2019	32	32	0	0.59	None	None	75%	NA	NA	^68^Ga-PSMA-PET/CT
Farolfi et al.	2019	119	119	0	0.32	0.5	34.40%	30.20%	NA	NA	^68^Ga-PSMA-PET/CT
Fendler et al.	2020	166	166	0	1.86	None	73% (not stratified)	65.66%	37.95%	27.71%	^68^Ga-PSMA-PET CT or MRI
Fendler et al.	2020	115	115	115	1.86	None	73% (not stratified)	66.95%	57.39%	9.56%	^68^Ga-PSMA-PET CT or MRI
Fendler et al.	2020	101	0	101	1.86	None	73% (not stratified)	73.27%	46.53%	26.70%	^68^Ga-PSMA-PET CT or MRI
Grubmuller et al.	2018	117	117	0	1.04	None	85.50%	42.70%	NA	NA	^68^Ga-PSMA-PET CT or MRI
Habl et al.	2017	100	100	0	0.69	None	76%	59.00%	NA	NA	^68^Ga-PSMA-PET CT or MRI
Hope et al.	2017	117	76 (33 received RT as well)	41	5.9 (mean)	None	82%	61%	53.20%	6.40%	^68^Ga-PSMA-PET CT or MRI
Huits et al.	2020	100	100	0	0.49	None	68%	68%	24%	44%	^68^Ga-PSMA-PET CT or MRI
Joshi et al.	2020	30	23	7	0.69	None	70.00%	70%	NA	NA	^68^Ga-PSMA-PET/MRI
Liu et al.	2020	79	0	79	4.8	None	87%	43%	NA	NA	^18^F-DCFPyL PET/CT
Meijer et al.	2021	150	150	0	0.5	None	70.70%	44%	NA	NA	^18^F-DCFPyL PET/CT
Meijer et al.	2021	41	41 (RP + SRT)	0	0.9	None	29.30%	31.70%	NA	NA	^18^F-DCFPyL PET/CT
Meijer et al.	2021	62	0	62	2.8	None	85.40%	38.70%	NA	NA	^18^F-DCFPyL PET/CT
Mena et al.	2017	68	59 (9 also received RT)	9	NA	None	60.30%	56.70%	NA	NA	^8^F–DCFBC PET/CT
Rousseau et al.	2019	52	52 (24 also received RT)	0	0.44	1.5	73.10%	73.10%	NA	NA	^68^Ga-PSMA-PET/CT
Schmidt-Hegemann et al.	2019	62	62	0	0.44	None	50%	50%	NA	NA	^68^Ga-PSMA-PET/CT
Tan et al.	2019	55	55	0	2.19	None	80%	56.80%	NA	NA	^68^Ga-PSMA-PET/CT
Van Leeuwen et al.	2015	70	70	0	0.2	1	54.30%	28.60%	NA	NA	^68^Ga-PSMA-PET/CT
Zacho et al.	2018	70	64	6	0.55	None	53%	43.50%	NA	NA	^68^Ga-PSMA-PET/CT

Abbreviations: RP, radical prostatectomy; RT, radiotherapy; PSA, prostate-specific antigen; PSMA, prostate-specific membrane antigen. NA: not applicable. ** 30% was for lesions found outside standard radiation pelvis fields not for treatment modification.

**Table 2:** Selected studies investigating the use of metastasis-directed therapy in patients with oligometastatic prostate cancer staged with PSMA PET.

Lead Author	Year	N	CS	CR	Concurrent ADT	Type of Study	Median PSA (ng/mL)	ADTFS/TEFS	PFS	Biochemical Response	Equipment and Radiotracer	Number of Lesions	Type of Patients
Artigas et al.	2019	20	20	0	0	Retrospective	1.4	ADTFS: 74% at 2 years	NA	70% at 4 months (decrease of >50% of PSA)	^68^Ga-PSMA-PET/CT	≤3 on Ga PSMA-PET	BCR after primary treatment
Guler et al.	2018	23	13	10	10	Retrospective	1.1	NA	67% for CS and 0% for CR at 1 year	NA	^68^Ga-PSMA-PET/CT	≤3 on Ga PSMA-PET	BCR after primary treatment
Phillips et al.	2020	SBRT 36	36	0	0	Prospective	6	NA	~58% at 24 months	NA	^18^F-DCFPyL PET/CT	≤3 on conventional imaging	BCR after primary treatment
Phillips et al.	2020	Observation: 18	18	0	0	Prospective	7	NA	0% at 24 months	NA	^18^F-DCFPyL PET/CT	≤3 on conventional imaging	BCR after primary treatment
Kalinauskaite et al.	2020	50	35	15	15	Retrospective	1.9	ADTFS: 60.5% at 2 years; TFFS: Median reached at 12 months	Median PFS reached at 12 months	NA	^68^Ga-PSMA-PET/CT	≤5 on PSMA-PET	Synchronous and metachronous OMPC
Hurmuz et al.	2020	176	NA	NA	140	Retrospective	18	NA	63.1% at 2 years	NA	^68^Ga-PSMA-PET/CT	≤5 on Ga PSMA-PET	Synchronous and metachronous OMPC
Oehus et al.	2020	78	NA	NA	13	Retrospective	1.9	Median 34 months (estimated; median not reached)	Median 17 months	Decrease from 1.90 to 0.88 at last follow-up	^68^Ga-PSMA-PET CT or MRI	≤5 on Ga PSMA-PET	Patients with biochemical progression after initial RP + SRT
Kneebone et al.	2018	57	57	0	0	Prospective	2.12 (mean)	NA	Mean bDFS at 11 months	NA	PSMA PET but specific radiotracer not specified	≤3 on PSMA-PET	BCR after primary treatment
Bowden et al.	2019	199	185	14	14	Prospective	1.8	TEFS: Median 27.1 months	NA	Decline below baseline in 75% of patients and persistent PSA below baseline in 23.3% at last follow-up	PSMA PET CT but specific radiotracer not specified	≤5 on imaging	BCR after primary treatment
Deijen et al.	2021	PSMA: 40	40	0	0	Retrospective	1.8	Median 32.7 months	NA	NA	^68^Ga-PSMA-PET/CT	≤4 on PSMA-PET or choline PET	Patients with oligometastatic (≤4 metastases) recurrent prostate cancer
Deijen et al.	2021	Choline PET: 10	10	0	0	Retrospective	4.2	Median 14.9 months	NA	NA	^68^Ga-PSMA-PET/CT	≤4 on PSMA-PET or choline PET	Patients with oligometastatic (≤4 metastases) recurrent prostate cancer
Onal et al.	2021	67	0	67	NA	Retrospective	3.5	Median 16.4 months	34.4% at 2 years	NA	^68^Ga-PSMA-PET/CT	≤5 on Ga PSMA-PET	CRPC

Abbreviations: CS, castration sensitive; CR, castration resistant; SBRT, stereotactic body radiotherapy; ADT, androgen deprivation therapy; PSA, prostate-specific antigen; PSMA, prostate-specific membrane antigen; ADTFS, androgen deprivation therapy—free survival; TEFS, treatment-escalation–free survival; PFS, progression-free survival; bDFS, biochemical-disease-free survival; BCR, biochemical recurrence; NA: not applicable; SRT, salvage radiotherapy; RP, radical prostatectomy; OMPC, oligometastatic prostate cancer.

**Table 3:** Summary of relevant clinical trials using PSMA PET for patients with prostate cancer.

Title	Identification Number	Number of Patients	Stage(s) of Disease	Randomized vs. Single Arm (R vs. S)	Relevant Endpoints	Radiotracer
Metastasis Directed Stereotactic Body Radiotherapy for Oligo Metastatic Hormone Sensitive Prostate Cancer (METRO)	NCT04983095	114	• Patients with CSPC with oligometastatic disease detected by PSMA-PET, including de novo oligometastatic CSPC and recurrent CSPC after primary RT or prostatectomy.	R (SBRT vs. standard treatment)	Failure-free survival; time to CRPC; OS	Not specified
An Investigational Scan (rh PSMA 7.3 PET/MRI) for the Detection of Recurrent Disease and Aid in Radiotherapy Planning in Biochemically Recurrent Prostate Cancer	NCT04978675	25	• Biochemically Recurrent Prostate Carcinoma after surgery.	S	Positive predictive value of rh PSMA 7.3 PET/MRI in detecting recurrent prostate cancer; change in radiation planning	Fluorine F 18 rhPSMA-7.3
The Role of 68Ga-PSMA-11 PET in Surgery Guidance in Prostate Cancer	NCT04936334	50	Men with NCCN unfavorable intermediate-, high- or very-high-risk prostate cancer scheduled for prostatectomy	S	PSMA PET predictive performance for detecting extra-prostatic extension; rate of change in management	68Ga-PSMA-11 PET
Apalutamide With or Without Stereotactic Body Radiation Therapy in Treating Participants With Castration-Resistant Prostate cancer (PILLAR)	NCT03503344	60	Patients with progressive CRPC during ADT, with at least one but no more than five discrete PSMA-avid lesions amenable to SBRT	R (Apalutamide vs. Apalutamide plus SBRT)	Proportion of patients with an undetectable PSA at 6 months; PSA progression; safety of apalutamide and SBRT.	68Ga-PSMA-11 PET
68Ga-PSMA-11 PET/CT for the Diagnosis of Bone Metastases in Patients With Prostate Cancer and Biochemical Progression During Androgen Deprivation Therapy	NCT4928820	102	Patient with biochemical progression during ADT or combination therapies, including ADT, who are referred for imaging evaluation (PSA > = 1 ng/mL that has increased on at least 2 successive occasions at least 1 week apart)	S	Detection rate relative to bone scan/CT; PFS; OS	68Ga-PSMA-11
Comparing Salvage Radiotherapy and Individualized PSMA PET/CT Targeted Treatment in With Relapsing Prostate Cancer	NCT04794777	450	Biochemical recurrence following surgery	R (standard salvage RT vs. individualizing therapy based on PET results)	PFS; time to metastases; time to secondary treatment	68Ga-PSMA-11 or 18F-PSMA-1007 PET/CT
Multicenter Randomized Trial of 68Ga-PSMA-11 PET/CT Based Salvage RT After Radical Prostatectomy	NCT03582774	193	Recurrence following primary prostatectomy	R (Standard salvage RT vs. individualizing therapy based on PET results)	Biochemical PFS; metastasis-free survival; initiation of additional salvage therapy; change in initial treatment intent	68Ga-PSMA-11
An Investigational Scan (68Ga-PSMA-11 PET/CT) for the Imaging of Prostate Cancer	NCT04777071	150	• Patients undergoing initial staging; • Biochemical recurrence after initial therapy; • Patients undergoing systemic therapy.	S	Change in planned management strategy	68Ga-PSMA-11
68 Ga-PSMA for High-Risk Prostate Cancer	NCT04614363	80	• High-risk untreated prostate cancer; • Biochemical recurrence after initial therapy.	S	Proportion of patients with lymph node involvement or in which planned clinical management was altered	68Ga-PSMA-11
PSMA PET/CT Guided Intensification of Therapy in Patients at Risk of Advanced Prostate Cancer (PATRON)	NCT04557501	776	• Patients with high-risk prostate cancer planned for primary treatment; • Patients with biochemical recurrence after surgery planned for salvage radiation.	R (treatment per standard of care vs. treatment adjusted per PET results)	Failure-free survival; Time to subsequent next-line therapy	Not specified
Randomized Trial of PSMA PET Scan Before Definitive Radiation Therapy for Prostate Cancer (PSMA-dRT)	NCT04457245	312	• Unfavorable intermediate-to-high-risk disease.	R (standard radiotherapy vs. radiotherapy planning per PET findings)	PFS; metastasis-free survival; OS; change in initial treatment intent	18F-DCFPyL
Metastasis-directed Therapy in Castration-refractory Prostate Cancer (MEDCARE)	NCT04222634	18	• Oligoprogressive mCRPC patients who will receive MDT.	S	Next-line systemic treatment-free survival	18F PSMA
68Ga-PSMA-11 PET/CT scan in Impacting Treatment Strategies for Patients with Prostate Cancer	NCT04050215	937	• Patients undergoing initial staging; • Patients undergoing restaging for recurrence post radiation or surgery or rising PSA in metastatic cancer.	S	Intended and implemented management changes	68GA-PSMA-11 PET/CT
PSMA PET/CT for Assessment of Recurrent Prostate Cancer	NCT02899312	1574	• Patients with biochemical recurrence after initial treatment; • CRPC with a minimum PSA of 2.0 ng/mL with 2 consecutive rises above the nadir and castrate levels of testosterone.	S	Superiority of PSMA PET over conventional imaging for detection of recurrence; clinical impact of PSMA-PET in patient management.	18F-DCFPyL

Abbreviations: SBRT, stereotactic body radiotherapy; RP, radical prostatectomy; RT, radiotherapy; PSA, prostate-specific antigen; PSMA PET, prostate-specific membrane antigen positron emission tomography; CT, computed tomography; MRI, magnetic resonance imaging; OS, overall survival; CSPC, castration-sensitive prostate cancer; CRPC, castration-resistant prostate cancer; ADT, androgen deprivation therapy; PSA, prostate-specific antigen; PFS, progression-free survival; MDT, metastasis-directed therapy.
